# Pulmonary Cusp Positioning of a Right Ventricular Outflow Tract Ventricular Tachycardia in a Pediatric Patient Identified Using Intracardiac Echocardiography

**DOI:** 10.19102/icrm.2020.110605

**Published:** 2020-06-15

**Authors:** Brian K. Lee, Anthony C. McCanta, Anjan S. Batra

**Affiliations:** ^1^Advocate Children’s Heart Institute, Advocate Children’s Hospital, Oak Lawn, IL, USA; ^2^Children’s Hospital of Orange County, Orange, CA, USA; ^3^University of California Irvine School of Medicine, Irvine, CA, USA

**Keywords:** Intracardiac echocardiography, nonsustained ventricular tachycardia, premature ventricular contractions, radiofrequency ablation, right ventricular outflow tract

## Abstract

Ventricular premature beats originating from the right ventricular outflow tract can have myocardial extensions to the pulmonary valve and pulmonary artery. Treatment may consist of catheter ablation combined with the use of three-dimensional mapping to determine the exact location of ectopy. The location of ectopy relative to the pulmonary valve may be hard to ascertain. Intracardiac echocardiography (ICE) is a noninvasive approach by which one can determine the relationship of the pulmonary valve relative to the ablation catheter prior to ablation. ICE has achieved increasing popularity during the ablation of other arrhythmias such as tricuspid valve arrhythmias and has been shown to be helpful in guiding catheter placement prior to ablation. The additional information gained from deploying ICE may ensure more precise ablation, prevent theoretical damage to the pulmonary valve, and alleviate the need for a repeat procedure. Here, we present a case involving the use of ICE during a pediatric patient’s second ablation procedure to precisely determine the location of ectopy of nonsustained ventricular tachycardia originating from the distal pulmonary valve.

## Introduction

Ventricular premature beats (VPBs) originating from the right ventricular outflow tract (RVOT) can have myocardial extensions to the pulmonary valve and pulmonary artery.^1^ Treatments for persistent symptoms include medical management and catheter ablation.^2^ Although three-dimensional mapping is routinely used in radiofrequency (RF) ablation procedures to determine the exact location of ectopy, the location relative to the pulmonary valve may be difficult to ascertain. Intracardiac echocardiography (ICE) is a noninvasive way to determine the relationship of the pulmonary valve relative to the RF catheter prior to ablation in real time without leaving the electrophysiology (EP) laboratory. ICE has experienced increasing popularity in the ablation of other arrhythmias, like the tricuspid valve arrhythmias, and has been shown to be helpful in guiding catheter placement prior to ablation.^3,4^ ICE also has a role in enhancing the safety of the ablation of RVOT arrhythmias near the pulmonary valve to prevent pulmonary valve dysfunction. Although not previously reported, inadvertent ablation energy applied to the pulmonary valve may impact its function. The additional information gained from using ICE may prevent the need for a repeat procedure. We herein present a case that incorporated ICE in a pediatric patient’s second RF ablation procedure to determine the precise location of ectopy of nonsustained ventricular tachycardia (NSVT) originating from the distal pulmonary valve.

## Case presentation

A 15-year-old female arrived at the Children’s Hospital of Orange County with exertional chest discomfort and palpitations. She already had undergone an EP study and RF ablation in the RVOT at another center. Although this prior ablation was acutely successful, she showed a very early recurrence of VPBs and rare symptoms of dizziness and shortness of breath. Her workup revealed ventricular bigeminy that suggested an RVOT etiology. Despite taking atenolol, she remained symptomatic. Her echocardiogram indicated normal cardiac function. Holter monitoring was performed twice, with resulting rates of monomorphic VPBs of 20.7% and 26.8%, respectively. The patient also had experienced a symptomatic event of syncope immediately after sprinting at basketball practice and was referred for exercise testing. During exercise testing, she showed frequent monomorphic VPBs that were suppressed with exercise. During recovery, she presented an abrupt onset of VPBs with the same morphology and symptomatic NSVT with dizziness and near-syncope. The decision was made to proceed with another EP study based on her persistent symptoms and a second possible RF ablation.

**Figure 1:**
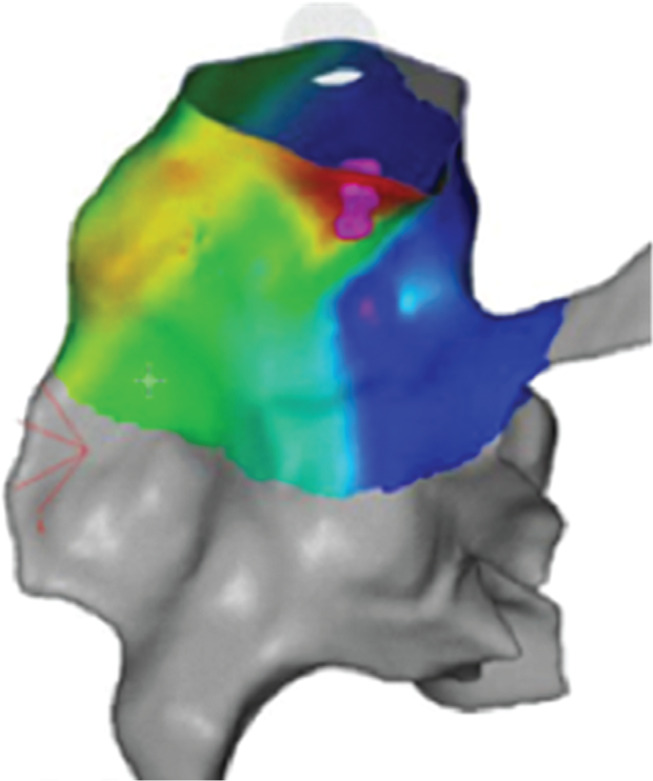
Three-dimensional electroanatomical activation map of VPBs with the posterior view showing early activation in the distal posterior RVOT.

**Figure 2:**
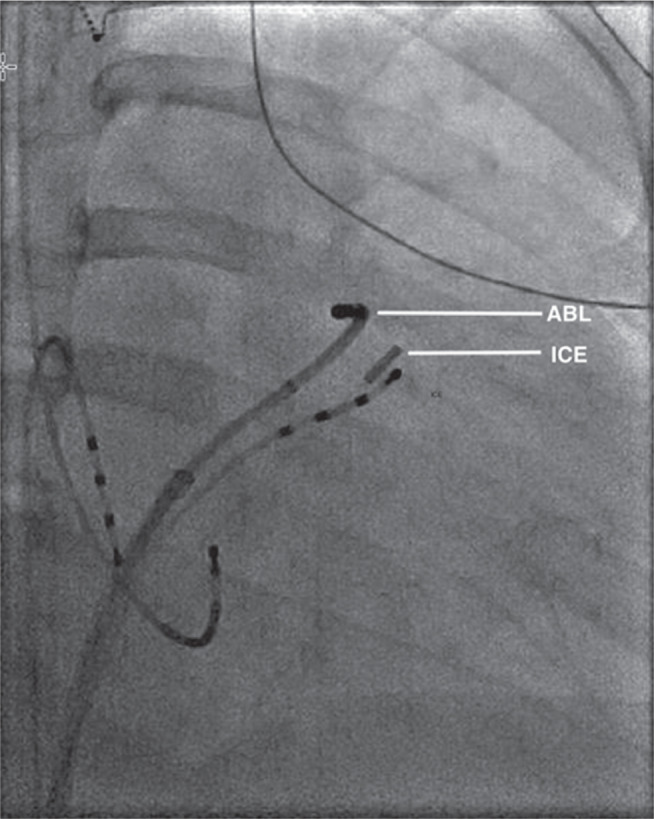
Biplane fluoroscopy image (right anterior oblique: 30 degrees) of the ablation catheter (ABL) positioned across the pulmonary valve, with the ICE catheter positioned in the proximal RVOT.

**Figure 3:**
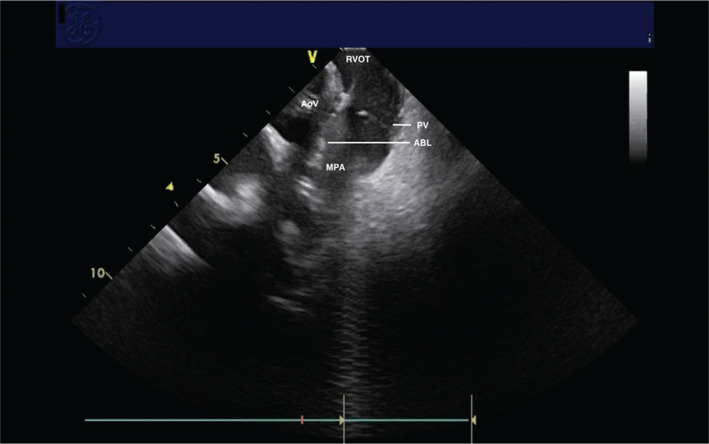
ICE image of the ablation catheter (ABL) across the pulmonary valve (PV) during RF ablation in the pulmonary cusp. The main pulmonary artery (MPA), aortic valve (AoV), and RVOT are also shown for reference.

**Figure 4:**
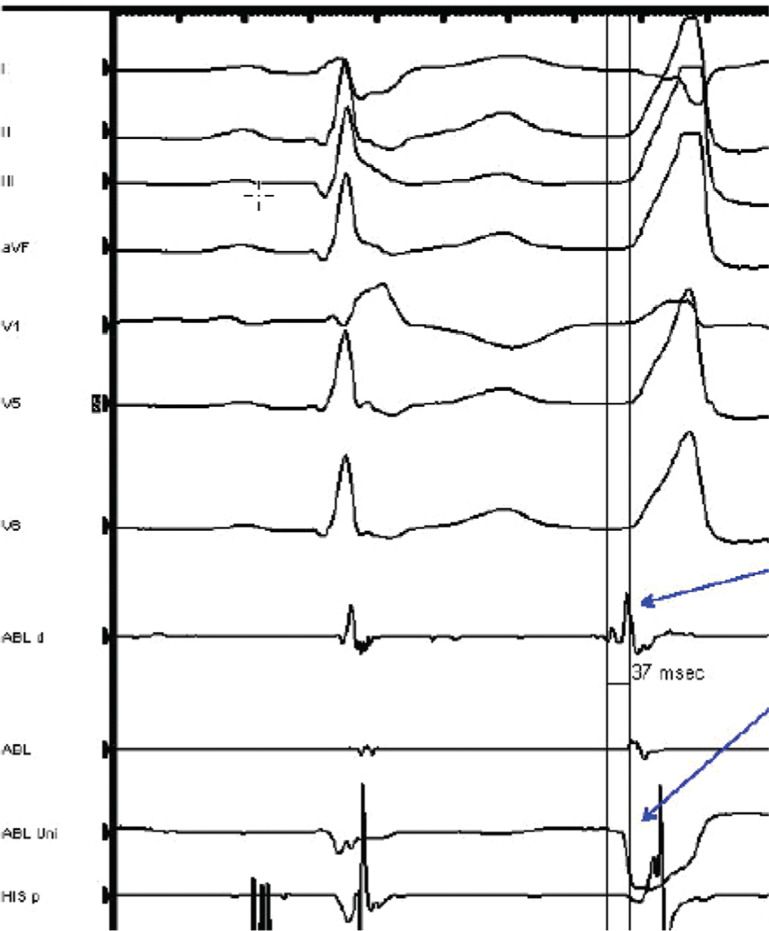
Intracardiac electrograms at the site of successful RF ablation in the pulmonary cusp. Early low-voltage bipolar activation preceded the surface QRS by 37 ms and pure Q-wave on unipolar recording. ABL d: ablation distal; ABL Uni: ablation unipolar.

During the EP study, three-dimensional electroanatomical mapping (CARTO^®^ 3; Biosense Webster, Diamond Bar, CA, USA) localized the VPBs to the RVOT **(Figure 1)**. The earliest bipolar activation in the RVOT was 20 ms presystolic, but unipolar electrograms from these locations had persistent R-waves. An 8-French (Fr) ICE catheter (Soundstar; Biosense Webster, Diamond Bar, CA, USA) was advanced to the proximal RVOT to visualize the location of the ablation catheter relative to the pulmonary valve **(Figure 2)**. The ablation catheter was then advanced across the pulmonary valve into an area within the pulmonary valve cusp in the left posterior aspect of the proximal pulmonary artery **(Figure 3)**. Presystolic activation was recorded at 37 ms earlier than the surface QRS with a pure Q-wave on the unipolar tracing **(Figure 4).** RF ablation with an irrigated-tip catheter (Thermacool; Biosense Webster, Diamond Bar, CA, USA) was performed with real-time visualization of the ablation catheter and its relationship to the pulmonary valve on ICE, which produced an acceleration of ventricular ectopy followed by the cessation of ventricular ectopic beats. The patient was asymptomatic during early follow-up, and repeat Holter monitoring revealed only six ectopic ventricular beats. One year after the procedure, she remained asymptomatic, with no VBPs observed during ambulatory monitoring.

## Discussion

Prior to the introduction of 8-Fr ICE catheters, ICE was not technically feasible in the majority of pediatric patients. The sizeable bore sheaths needed to introduce 10-, 12-, or 14-Fr ICE catheters were prohibitively too large for pediatric patients. However, in this case, the use of an 8-Fr ICE catheter advanced into the proximal RVOT successfully resulted in a real-time evaluation of the ablation catheter and its relationship to the pulmonary valve leaflets. Although the RVOT is a common origin for idiopathic VT and VPBs, the pulmonary cusp remains a somewhat rare site of origin^1^ even though the RVOT interdigitates with the same myocardial extensions that surround the aortic root and aortic cusp, which are common sites of ablation of left ventricular outflow tract tachycardias. Advancing the ablation catheter into the pulmonary cusp resulted in a dramatic improvement in bipolar and unipolar activation times, with a pure Q unipolar electrogram, and ablation at the site of these electrograms resulted in complete and long-lasting success for this patient.

Published success rates of RF ablation for VPBs from the RVOT have been excellent and range from 93% to 97.1%^5–7^; however, very few pediatric patients were included in these studies. Further, complication rates published from these investigations focused on acute complications like acute cardiac events or those related to vascular access. To our knowledge, there has been no study that assessed the long-term outcomes of RF ablation of VPBs from the RVOT. Although injury to the pulmonary valve has not been reported, direct visualization of the ablation catheter and its proximity to the pulmonary valve was not routinely conducted or documented. Thus, it is our recommendation to use ICE in conjunction with RF ablation to better visualize the pulmonary valve to avoid causing potential damage to the valve. In our patient, the ectopic focus was located in the pulmonary cusp and was successfully ablated without signs of pulmonary valve dysfunction during her immediate postoperative course. In addition, the use of ICE initially may have prevented the need to repeat the procedure, which carries a number of concerns including those associated with sedation, thus making the cost–risk ratio more favorable.

## Conclusion

We report on the use of an 8-Fr ICE catheter positioned in the RVOT to identify and successfully ablate a ventricular tachycardia originating from the pulmonary cusp in a pediatric patient with symptomatic ventricular tachycardia. The availability of smaller ICE catheters can help to expand the utility of ICE for pediatric patients through enhancing the safety of the procedure by minimizing the impact of vascular access.
